# Maintenance of distinct melanocyte populations in the interfollicular epidermis

**DOI:** 10.1111/pcmr.12375

**Published:** 2015-04-30

**Authors:** James D. Glover, Stefan Knolle, Kirsty L. Wells, Dianbo Liu, Ian J. Jackson, Richard L. Mort, Denis J. Headon

**Affiliations:** ^1^ The Roslin Institute and Royal (Dick) School of Veterinary StudiesUniversity of EdinburghRoslinUK; ^2^ MRC Human Genetics UnitMRC IGMMWestern General HospitalUniversity of EdinburghEdinburghUK; ^3^ Present address: Brandenburgische Technische Universität Cottbus‐SenftenbergSenftenbergGermany; ^4^ Present address: College of Life SciencesUniversity of DundeeDundeeUK

**Keywords:** epidermis, hair follicle, interfollicular, melanocyte, stem cell

## Abstract

Hair follicles and sweat glands are recognized as reservoirs of melanocyte stem cells (MSCs). Unlike differentiated melanocytes, undifferentiated MSCs do not produce melanin. They serve as a source of differentiated melanocytes for the hair follicle and contribute to the interfollicular epidermis upon wounding, exposure to ultraviolet irradiation or in remission from vitiligo, where repigmentation often spreads outwards from the hair follicles. It is unknown whether these observations reflect the normal homoeostatic mechanism of melanocyte renewal or whether unperturbed interfollicular epidermis can maintain a melanocyte population that is independent of the skin's appendages. Here, we show that mouse tail skin lacking appendages does maintain a stable melanocyte number, including a low frequency of amelanotic melanocytes, into adult life. Furthermore, we show that actively cycling differentiated melanocytes are present in postnatal skin, indicating that amelanotic melanocytes are not uniquely relied on for melanocyte homoeostasis.

**Significance**

The ability of interfollicular epidermis to carry a resident melanocyte population independently of the hair follicles indicates that cutaneous melanocyte populations are maintained by distinct mechanisms. This potential heterogeneity should be taken into account in research efforts to stimulate repigmentation and in addressing the origins and behaviours of melanomas.

During embryonic development, hair follicles are colonized by melanoblasts, the precursors of differentiated, pigment‐producing melanocytes. In the adult, hair follicles lose differentiated melanocytes at the end of each hair cycle and maintain quiescent, non‐pigment‐producing melanocytes in a region known as the bulge. These stem cells give rise to progeny which repopulate the hair bulb with active melanin‐producing melanocytes during each hair cycle. The stem cells can also be stimulated by wound healing, ultraviolet irradiation or ectopic expression of the survival factor Kit‐ligand to migrate to the interfollicular epidermis where they differentiate and transfer pigment to their surrounding keratinocytes (Chou et al., 2013; Nishimura, 2011; Nishimura et al., 2002). In humans, repigmentation in vitiligo is often observed to initiate from the hair follicles before spreading out to make a continuous colouring to the skin, supporting the idea that hair follicle‐derived melanocytes contribute functionally to interfollicular pigmentation (Yamaguchi and Hearing, 2014). A stem cell niche has also been identified in another cutaneous appendage, the eccrine sweat gland, which contains melanocytes with stem cell characteristics (Grichnik et al., 1996; Okamoto et al., 2014).

Although there is clear evidence that cutaneous appendages can provide melanocytes to the skin in specific situations, it is unknown whether melanocytes of the interfollicular epidermis normally have their ultimate source in the hair follicle. Nevertheless, regions of skin in patients with vitiligo that lack hair follicles, such as the palms of the hands, can occasionally become repopulated by melanocytes, and Li et al. (2010) have described cells derived in culture from neonatal dermis that can become melanocytes when in contact with epidermal keratinocytes. We report here that interfollicular epidermis is capable of long‐term maintenance of melanocytes, including a population of amelanotic (i.e. non‐melanin‐producing) melanocytes, in the absence of hair follicles. We also show that differentiated, melanin‐producing, melanocytes are capable of proliferation, suggesting that the unpigmented melanocytes observed may not be required for routine melanocyte production during growth or homoeostasis.

We addressed the relationship between hair follicle and interfollicular melanocyte populations in the mouse tail, a region which, as in human skin, has a pigmented interfollicular epidermis in addition to pigmented hairs. *Eda* is an X‐linked gene required for epidermal appendage development. We explored the functional relevance of hair follicles in pigmentation and melanocyte maintenance by comparing the characteristics of hemizygous *Eda* mutant males (*Eda* ^*Ta*/*Y*^), which lack development of hair follicles on the tail (Falconer, 1953), with those of their wild‐type male littermates (*Eda* ^*+/Y*^). In the absence of hair follicles, melanin production and transfer to keratinocytes occur as in wild type, where it is restricted to the scale plates and not the interscale hinges (Figure 1A). L‐DOPA staining reveals active melanin‐producing melanocytes in the epidermis of both wild‐type haired and mutant non‐hairy skin (Figure 1B), although overall melanocyte density is significantly greater in *Eda* ^*Ta/Y*^ skin than in wild type (Figure 1C), and this density is stable across all ages examined. The greater abundance of melanocytes in *Eda* ^*Ta/Y*^ skin is a result of their more widespread distribution compared with melanocytes in the wild type, which are restricted to the scales (Figure 1D) as revealed by X‐gal staining in the *Dct::lacZ* line (Mackenzie et al., 1997). Melanocyte density specifically in the scale regions, where their concentration is greater, is similar in wild‐type and *Eda* ^*Ta/Y*^ tail skin (Figure 1C).

**Figure 1:**
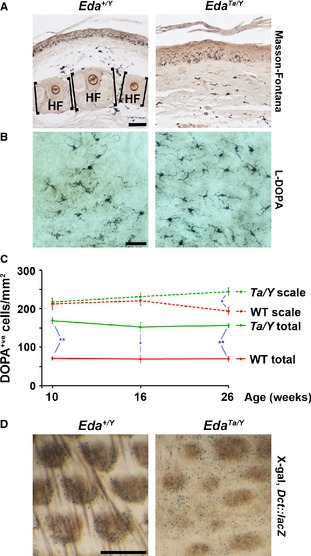
Functional melanocytes persist in the absence of hair follicles. (A) Detection of melanin by Masson‐Fontana stain in epidermis and dermis of wild‐type (*Eda* ^*+/Y*^) and mutant (*Eda* ^*Ta/Y*^) tail skin at 10 weeks. Scale bar 50 *μ*m. HF denotes hair follicles and associated sebaceous glands. (B) Detection of active melanocytes by L‐DOPA staining in wild‐type and mutant epidermis at 16 weeks. Scale bar 50 *μ*m. (C) Quantification of melanocyte density in all tail interfollicular epidermis and specifically in the scale regions through adult life in wild‐type (WT) and mutant (*Ta/Y*) mice. A stable melanocyte population is maintained in the mutant in the absence of hair follicles. Error bars represent SEM. Significant difference was calculated using a Student's t test * *P *<* *0.05, ** *P *<* *0.01. (D) Distribution of melanocytes in 4‐week‐old wild‐type and *Eda* ^*Ta/Y*^ epidermis, revealed by X‐gal staining (blue colour) of *Dct::lacZ* transgenic tissue. Scale bar 0.5 mm.

The undifferentiated melanocyte stem cells that reside in the hair follicle bulge do not produce melanin (Nishimura et al., 2002). We sought to determine whether an analogous population exists in the interfollicular epidermis. We generated animals carrying the *Tyr::Cre* transgene, which drives Cre‐mediated recombination in the entire melanocyte lineage (Delmas et al., 2003), and the *Rosa26::mT/mG* allele, which switches membrane fluorescence from red (tdTomato) to green (EGFP) in cells which have undergone Cre‐mediated recombination (Muzumdar et al., 2007). This allows visualization of all descendants of the melanoblast lineage as membrane EGFP‐labelled cells. As expected, two populations of melanocytes are readily detected in anagen hair follicles, at the hair bulb and the bulge, in isolated epidermis from this line (Figure 2A). We then stained epidermis from wild‐type or *Eda* ^*Ta/Y*^ mice carrying these transgenes with L‐DOPA to distinguish amelanotic melanocytes from pigment‐producing melanocytes. Positive L‐DOPA staining suppressed EGFP fluorescence, allowing ready identification of EGFP‐positive, L‐DOPA‐negative cells, which represent amelanotic melanocytes (Figure 2B). These amelanotic melanocytes in *Eda* ^*Ta/Y*^ skin could not have emerged from hair follicles or sweat glands at any stage of development as these appendages had never developed in the mutant skin.

**Figure 2:**
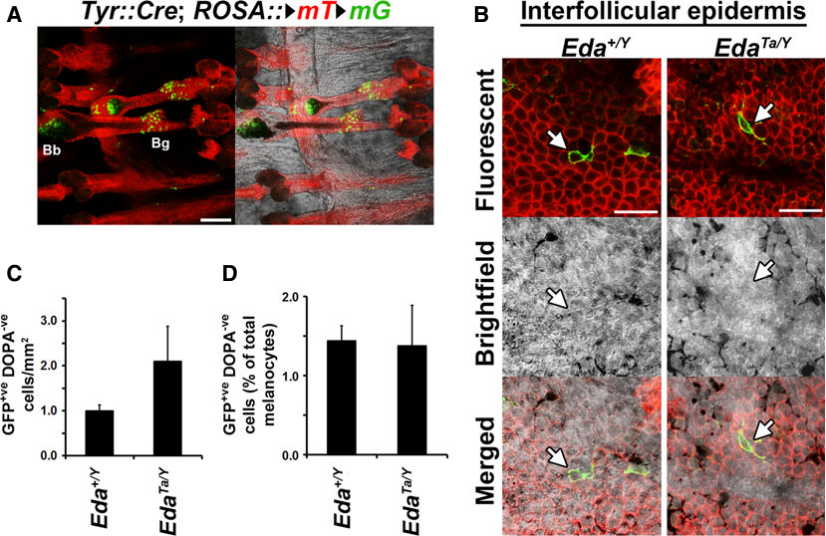
Amelanotic melanocytes exist in the interfollicular epidermis. (A) Isolated epidermis from *Tyr*::*Cre*;* Rosa26::mT/mG* at 16 weeks. Melanocytes express membrane‐EGFP, while other cells have membrane‐tdTomato. Left panel, fluorescence in red and green channels. Right panel, fluorescence merged with brightfield. Hair follicles have melanocyte populations at the bulb (Bb) and bulge (Bg). Scale bar 200 *μ*m. (B) L‐DOPA‐negative interfollicular epidermal melanocytes (indicated by arrows) are interspersed amongst the differentiated melanocytes in wild‐type (*Eda* ^*+/Y*^) and mutant (*Eda* ^*Ta/Y*^) isolated epidermis. Scale bar 25 *μ*m. (C, D) L‐DOPA‐negative melanocyte density in 16‐week‐old wild‐type and mutant interfollicular epidermis, by area (C) and as a percentage of total melanocytes (D). Error bars represent SEM.

We quantified the frequency of amelanotic melanocytes in the epidermis, finding similar values in haired and hairless (wild type and *Eda* ^*Ta*/*Y*^) skin of approximately 1.5% of the total melanocyte population (Figure 2C,D). In the hair follicle, keratinocyte stem cells and the amelanotic melanocyte stem cells coexist specifically in the same niche at the bulge (Nishimura, 2011), while in tail interfollicular epidermis, keratinocyte stem cells reside primarily in the interscales (Mascré et al., 2012). However, we identified amelanotic melanocytes in both interscale hinges and in the scales, interspersed amongst the pigment‐producing melanocytes. Thus, in contrast to the hair follicles, amelanotic melanocytes of the interfollicular epidermis do not strictly cohabit the same location as the keratinocyte stem cells.

To further investigate melanocyte homoeostasis, we crossed *Tyr::Cre* mice with *R26Fucci2aR* animals containing a Cre‐inducible bicistronic Fucci2a reporter to identify cell cycle stage in melanocytes (Mort et al., 2014). Recombination at this locus yields cells which express the cell cycle reporters mCherry‐hCdt1(30/120) (degraded in the S, G2 and M phases of the cell cycle) and mVenus‐hGem(1/110) (absent in late M, G1 and G0 phases) (Figure 3A). G1/G0 melanocytes are therefore red, and the S/G2/M fraction that is actively cycling is labelled green. The majority of interfollicular tail melanocytes at 8 weeks observed from this cross were mCherry positive (not shown) suggesting a low frequency of actively cycling cells. However, tail interfollicular epidermis between postnatal days 8 and 19, which is actively growing, contained distinct mVenus‐positive and mCherry‐positive melanocytes. Interestingly, in general, the mVenus‐positive, proliferative, melanocytes were dendritic and contained melanin (detectable with the transmitted light detector of the confocal microscope, Figure 3B). To substantiate the correspondence between cell cycle phase detected using the Fucci2a transgenic system and cell division itself, we live‐imaged explant cultures of *Tyr::Cre*;* R26Fucci2aR* skin at postnatal day 19. We found that cells displaying green fluorescence at the start of the culture went on to divide over the following hours (Figure 3C and Video S1) demonstrating in this system, as observed directly for melanocytes in zebrafish (Taylor et al., 2011) and as implied by tritiated thymidine uptake by L‐DOPA‐positive melanocytes in mouse skin (Nordlund et al., 1986; Sato and Kawada, 1972), that active melanin‐producing mammalian melanocytes are capable of proliferation *in vivo*. Imaging revealed that proliferating melanocytes contained pigment throughout the process of cell division, indicating that the amelanotic melanocytes that we detect are not simply at a different cell cycle phase than their neighbours but rather likely represent a stable population.

**Figure 3:**
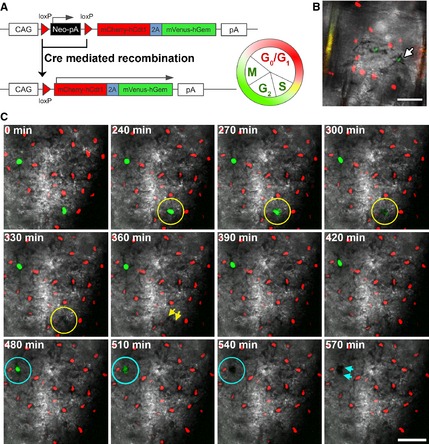
Melanin‐producing melanocytes are capable of cell division in mouse skin. (A) Schematic showing marking of cell cycle phase using the Fucci2a system. (B) Differentiated, melanin‐containing melanocytes in 9‐day‐old wild‐type mice carrying the *Tyr::Cre* transgene and *R26Fucci2aR* allele are found in the S/G2/M phases of the cell cycle (arrow). Scale bar 50 *μ*m. (C) Panels show time lapse of explant cultured *Tyr::Cre*;* R26Fucci2aR* skin at P19. Melanocytes in S/G2/M phases of the cell cycle (green fluorescent signal, indicated by circles) undergo division (arrows) without losing melanin pigment. Scale bar 50 *μ*m.

Embryonic skin is initially colonized by melanoblasts derived from the neural crest. A subset of these cells goes on to enter the developing hair follicles where they both pigment the first hair growth and form the stem cell population that will replenish the pigment‐producing melanocytes in subsequent hair cycles. In human skin, and some regions of mouse skin, interfollicular epidermal melanocytes also persist and pigment the surrounding keratinocytes. This study addresses whether hair follicles are an essential source of interfollicular melanocytes as the skin grows postnatally and into adulthood, and finds that they are not. We also observe that melanin‐producing melanocytes of the interfollicular epidermis are capable of proliferation in the postnatal period. In addition, we have identified an amelanotic population of melanocytes in the interfollicular epidermis distinct from the melanocyte stem cells of the hair follicle and sweat glands. These amelanotic melanocytes may serve as a reserve that is used for repopulation upon large‐scale melanocyte loss, analogous to the putative population identified in the interfollicular epidermis of both vitiliginous and healthy skin in humans (Seleit et al., 2014). Recognition of these distinct melanocyte cell populations, and their potential for different behaviours, will be important in understanding melanocyte population homoeostasis, its recovery following depletion, and melanomagenesis (Garcia et al., 2011).

## Supporting information


**Video S1.** Live imaging of cell cycle progression in differentiated melanocytes.
